# Tooth loss as a risk factor for dementia: systematic review and meta-analysis of 21 observational studies

**DOI:** 10.1186/s12888-018-1927-0

**Published:** 2018-10-20

**Authors:** Wen-li Fang, Mu-jun Jiang, Bei-bei Gu, Ying-mei Wei, Sheng-nuo Fan, Wang Liao, Yu-qiu Zheng, Shao-wei Liao, Ying Xiong, Yi Li, Song-hua Xiao, Jun Liu

**Affiliations:** 1 Department of Neurology, Sun Yat-sen Memorial Hospital, Sun Yat-sen University, 107 Yanjiang West Road, Guangzhou, 510120 Guangdong China; 2 Faculty of Pharmacy, Bengbu Medical College, Bengbu, Anhui China; 3 Department of Anesthesiology, Sun Yat-sen Memorial Hospital, Sun Yat-sen University, Guangzhou, Guangdong China; 4 Department of Neurology, The Seventh Affiliated Hospital of Sun Yat-sen University, Guangzhou, Guangdong China; 5 Laboratory of RNA and Major Diseases of Brain and Heart, Sun Yat-sen Memorial Hospital, Sun Yat-sen University, Guangzhou, Guangdong China; 6 Guangdong Province Key Laboratory of Brain Function and Disease, Zhongshan School of Medicine, Sun Yat-sen University, Guangzhou, Guangdong China

**Keywords:** Dementia, Cognitive impairment, Tooth loss, Risk assessment, Meta-analysis

## Abstract

**Background:**

Tooth loss is suggested to be associated with an increased risk of dementia in many studies. But the relationship between tooth loss and dementia is not yet fully understood. This systematic review and meta-analysis aimed to determine the relative effect of tooth loss on dementia risk.

**Methods:**

An electronic search of PubMed, Scopus, Embase, and Web of Knowledge was conducted in March 2018 to identify relevant observational studies with the English language restriction. Studies were included if they assessed the relationship between tooth loss and risk of dementia. Study quality was detected by the modified Downs and Black scale. Odds risks (ORs) were pooled using a random-effects model in the crude model.

**Results:**

The literature search initially yielded 1574 articles, and 21 observational studies published between 1994 and 2017 were finally included for the analyses. The crude results with random-effects model showed that patients with multiple tooth loss had higher incidence of dementia (OR 2.62, 95% CI 1.90–3.61, *P* < 0.001, *I*^*2*^ = 90.40%). The association remained noted when only adjusted results were pooled from 18 studies (OR 1.55, 95% CI 1.41–1.70, *P* = 0.13, *I*^*2*^ = 28.00%). Meta-regression analysis showed that study design explained about 16.52% of heterogeneity in the crude model. The overall quality rating scores of studies ranged from 11 to 16.

**Conclusions:**

Findings from this review evidenced that tooth loss is positively associated with an increased risk of dementia in adults. Future well-designed longitudinal researches examining the direct and indirect relationship between tooth loss and dementia risk are encouraged.

**Electronic supplementary material:**

The online version of this article (10.1186/s12888-018-1927-0) contains supplementary material, which is available to authorized users.

## Background

Dementia is characterized by cognitive and functional decline and neuropsychiatric symptoms caused by irreversible neurodegenerative diseases. The global population is aging at a rapid pace due to rising life expectance and over 47 million people live with dementia in 2016. The prevalence of dementia results in negative impacts on people’s life quality and economy according to the 2016 World Alzheimer Report [1]. To our knowledge, there is no effective anti-dementia drug available for the management of dementia. Therefore, it is in great need to identify modifiable risk factors for preventing cognitive impairment.

Tooth loss is prevalent in patients with dementia and it is a worldwide public health issue in older adults [2], impacting negatively on their quality of daily life, such as chewing, swallowing, and social life [3–5]. Evidence has shown that tooth loss is not only associated with oral health, but also with systemic health [6]. Recently, increasing studies have focused on the link between tooth loss and the risk of dementia [7–12]. There are several potential mechanisms by which tooth loss can negatively impact cognitive function. Periodontitis is one of the main causes of tooth loss, which is able to increased levels of pro-inflammatory mediators such as IL-1, IL-6 and TNF-α in the plasma, contributing to the aggravation of neuroinflammatory processes in brain and eventually resulting in cognitive decline [13–15]. Besides, masticatory disorder due to tooth loss can lead to poor nutrition, and reduce cerebral blood flow, which may be linked to memory deficits [9, 10]. It has been supported by several animal studies that tooth loss may induce decreased acetylcholine levels due to masticatory dysfunction, and lead to reductions in the number of pyramidal cells in the hippocampus, provoking cognitive dysfunction [16, 17].

A growing number of primary studies have demonstrated a close relationship between tooth loss and incidence of dementia, suggesting that tooth loss may be a modifiable risk factor for dementia [18–27]. However, this association is not noted in some studies [28–38]. To our knowledge, there are only two limited meta-analysis released by Shen et al [39] and Oh et al [40], exploring the relationship between tooth loss and cognitive impairment. In fact, some vital studies were not included without clear reasons, although Shen and colleagues have included observational studies from different study designs in the review. Moreover, the flow diagram of identification and selection process of studies could not be found in the analysis [39, 40]. Additionally, qualitative evaluation of selected studies and confounders for adjusted results of included studies were not demonstrated in the paper. As for the meta-analysis by Oh and colleagues, they intended to include cohort studies to prevent significant selection bias from cross-sectional studies [40]. However, one of the included studies is a cross-sectional design study, which was released by Luo et al [18]. Based on that, we therefore conducted a well-designed systematic review and meta-analysis of observational studies describing the association between tooth loss and the incidence of dementia in adults. We hope that our results can shed some light on the prevention of dementia in the future.

## Methods

### Search strategy

We systematically searched electronic databases, including PubMed, EMBASE, Scopus and Web of Science, to identify studies that analyzed the association between tooth loss and dementia in adults from inception to March 2018 with the English language restriction using the key terms: dementia, Alzheimer’s, mild cognitive impairment, cognitive impairment, cognitive decline, cognitive disorder, memory disorder, memory disorder, tooth loss, oral health and dental care. References of relevant papers were also screened for additional publications and we did not retrieve unpublished studies. Predefined data-collection worksheets were employed for the assessment of each included paper. Any disagreement among authors was resolved by discussion until a consensus was reached.

### Inclusion/exclusion criteria

For inclusion in this analysis, eligible studies should define tooth loss as one of the exposure interests, while incidence of dementia as one of the outcome of interests, and present original data or an crude and/or adjusted effect size, such as odds ratio (OR), hazard ratio (HR), or risk ratio (RR) of dementia with their 95% confidence intervals (CIs), or enough data to quantify the association between tooth loss and dementia risk. Different study designs were included. Abstracts from conferences, letters to the editor and reviews were excluded in the overall analysis. Animal studies were also excluded in this analysis. Moreover, concerning the quality assessment criteria, studies with a quality score of less than 5 points were not considered.

### Quality assessment

The quality of all selected studies was assessed using an adaptation of the Downs and Black criteria as described in previous systematic reviews [41–43]. From 27 original items in the checklist of the Downs and Black criteria, 17 were employed to accommodate the characteristics of observational studies, while other items specific for interventional randomization studies were removed. As recommended by Wehrmeister and colleagues [44], the total scores range from 0 to 18 points, given that each item scores one point, except for item 4 that can result in 0 (no), 1 (partially) and 2 (yes). Studies could be categorized with a quality score as: high chance of bias (0–5 points), moderate chance of bias (6–11 points) and low chance of bias (12–18 points) [41]. Two reviewers rated each study independently according to the above quality criteria, and discrepancies were discussed and resolved by consensus between referees.

### Data extraction

We extracted data independently from each included study, using a standardized worksheet in particular concerning: name of first author, publication year, study region, study design, age, sample size, main exposures definition, crude effect size with their 95%CI, adjusted effect size with their 95%CI, and adjusted variables, follow-up time. We extracted the highest versus lowest effect size with their 95%CI of tooth loss number associated with dementia incidence for this analysis. The effect sizes with their 95%CI adjusted with the most confounders were extracted for the adjusted model [39]. Disagreements of methodology or result between investigators were solved by consensus.

### Statistical analysis

The publications reported different measures of estimate effects including RR, OR and HR with their 95%CIs. Based on the assumption that the absolute risk of dementia was low and the person time of the exposed group was much smaller than that of the unexposed group, we did not make distinction between these size effects in this study. This way of pooling different measures of estimate effects has been used previously [45–49]. Meta-analyses were performed considering crude correlation between tooth loss and dementia risk and adjusted association between tooth loss and dementia risk. When various categories of tooth loss were shown, only the estimate comparing the most extreme categories was used for analysis as described in previous Meta-analyses [39, 41] Heterogeneity among studies was quantified using the Cochran’s *Q* test and chi-square (*I*^*2*^) test. Heterogeneity was considered statistically significant with *P* < 0.05 and random-effects model was used when heterogeneity was obvious *(I*^*2*^ > 50%) in this meta-analysis. Subgroup analyses and meta-regression were performed to explore the source of heterogeneity and it was conducted by the following subsets: study design (case-control or cohort or cross-sectional study), sample size, study region, and cognitive assessment. These approaches helped to identify whether the study characteristics mentioned above statistically affected estimate effects. We also assessed publication bias using both Begg-Mazumdar test and Egger’s regression test. When significant bias was found, we performed the trim and fill method to adjust for it. All analyses were completed with the Meta-analysis program software STATA 12.0 (StataCorp, College Station, TX, USA).

## Results

**Fig. 1:**
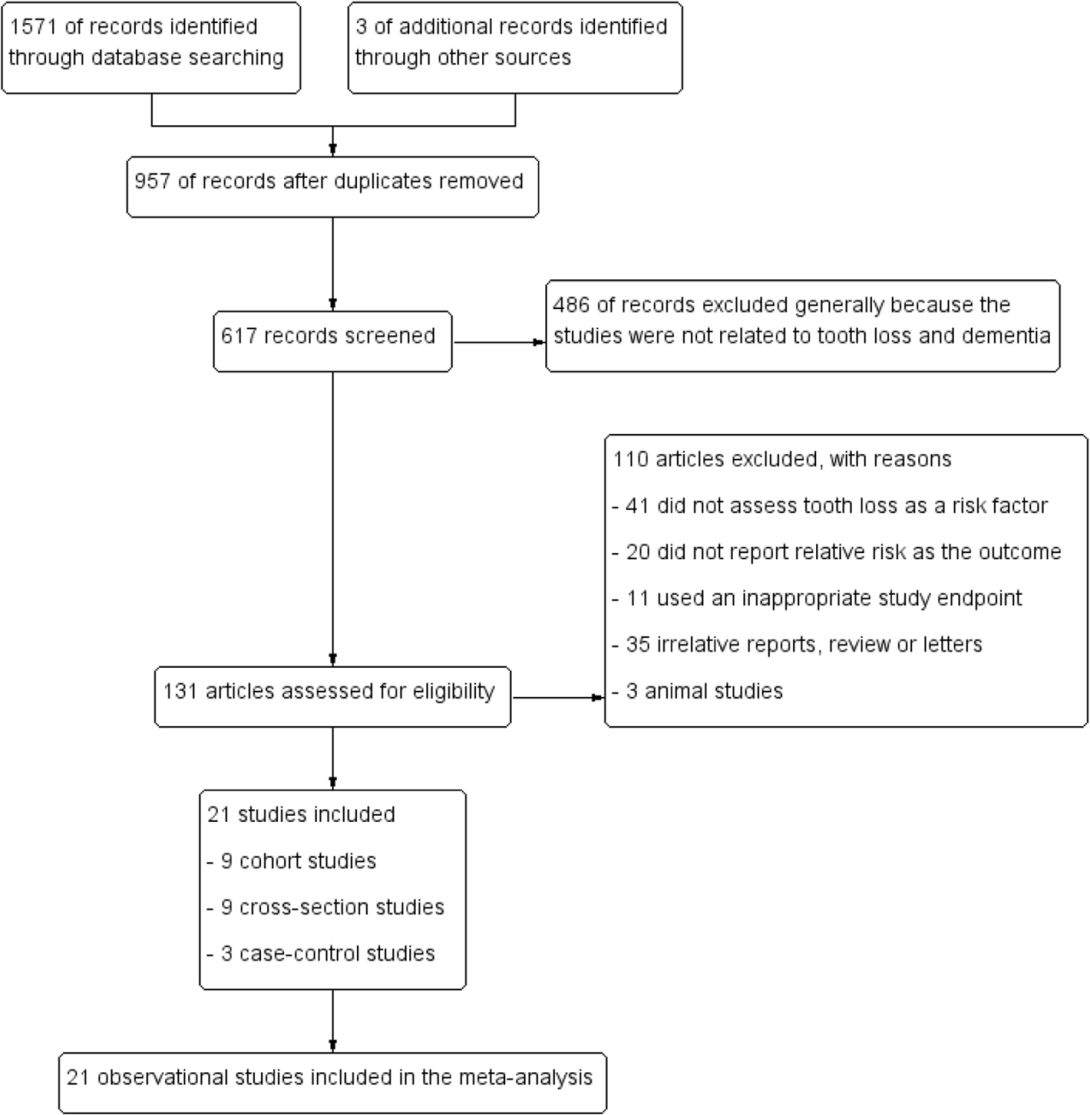
Flow diagram of identification and selection process of studies

The selecting processes for eligible studies were shown in Fig. 1. The literature search initially yielded 1574 papers, and 957 studies were duplicated. Abstracts from conferences, letters to the editor and reviews were excluded. Articles only with animal experiments or with repetitive data were removed. In addition, studies failed to provide enough data to quantify the association between tooth loss and dementia risk were also excluded. Finally, 21 studies published between 1994 and 2017 were identified for this analysis. Among all the studies, there were nine cross-sectional studies, nine cohort studies and three case-control studies and all the included studies were published in English. The main characteristics of studies were described in Tables 1, 2 and 3. Among these studies, nine were carried out in Asia, six in Europe and three in America. The total quality rating scores of included studies ranged from 11 to 16.

**Table 1:** Summary of cross-sectional studies included in the meta-analysis

Author /Year	Country	Sample size	Study design	Age, yr	Main exposure definition	Exposure cut-off point	Accessment of cognitive function	Effect size and crude association results with 95%CI highest vs. lowest category	Effect size and adjusted association results with 95%CI highest vs. lowest category	Adjustment	Quality scores
Luo et al (2015) [18]	China	3063	Cross sectional	≥60	Number of teeth missing	0–3, 4–6, 7–16, > 16	DSM-IV	3.65 (2.75–4.86)	1.56 (1.12–2.18)	Sex, age, education year, living alone, overweight, cigarette smoking, alcohol drinking, anxiety, depression, heart disease, hypertension, diabetes, and Apolipoprotein E-ε4	15
Peres et al (2014) [19]	Brazil	1705	Cross sectional	≥60	Number of teeth present	≥10, < 10, 0	MMSE	6.40 (3.40–12.10)	3.30 (1.20–9.30)	Sex, age, race, income, education, smoking, depression, diabetes, cardiova-scular disease, and hypertension	14
Nilsson et al (2014) [20]	Sweden	1147	Cross sectional	60–96	Number of teeth present	≥20, 1–19, 0	MMSE	9.20 (5.90–14.30)	3.20 (1.90–53.00)	Age and education	15
Wang et al (2014) [28]	China	930	Cross sectional	≥65	Number of teeth present	≥20, <20	MMSE	1.54(1.13–2.10)	1.30 (0.93–1.81)	Age, gender and life style habits	14
Park et al (2013) [21]	Korea	438	Cross sectional	≥50	Number of teeth missing	0–5, 6–10, > 10	MMSE	2.69(1.57–4.64)	2.25 (1.26–4.02)	Age, gender, education, hypertension, diabetes, hyperlipidemia and current smoking	13
Saito et al (2013) [22]	Japan	462	Cross sectional	≥60	Number of teeth present	22–32, 11–21, 0–10	MMSE	27.33(3.62–206.21)	20.21 (2.20–185.47)	Age, gender, education, smoking, alcohol intake, positive history of diseases, TMIG-IC score, and CES-D total score	13
Lexomboon et al (2012) [29]	Sweden	557	Cross sectional	≥77	Number of teeth missing	Multiple tooth loss	MMSE	2.10 (1.35–3.25)	1.36 (0.84–2.19)	Sex, age, and education	12
Okamoto et al (2010) [23]	Japan	4061	Cross sectional	≥65	Number of teeth present	22–32, 11–21, 0–10	MMSE	–	2.18 (1.51–3.14)	Depressive symptoms, age, sex, length of education, frequency of drinking, smoking habit, time spent walking every day, positive history of cancer and diabetes mellitus, and the levels of serum albumin, total cholesterol, and low-density lipoprotein cholesterol	12
Stewart et al (2007) [24]	England	4032	Cross sectional	≥65	Number of teeth present	> 0, 0	AMTS	3.59 (2.36–5.47)	2.61 (1.49–4.28)	Age, sex, education, sampling area, disability, and BMI	12

Note: *CI* Confidence interval, *AMTS* Abbreviated Mental Test Score, *TMIG-IC* The Tokyo Metropolitan Institute of Gerontology Index of Competence, *CES-D* The Center for Epidemiologic studies depression scale, *BMI* Body mass index, *MMSE* Mini-mental status examination, *DSM-IV* The Diagnostic and Statistical Manual of Mental Disorders Fourth Edition

**Table 2:** Summary of cohort studies included in the meta-analysis

Author /Year	Country	Sample size	Study design	Age, yr	Main exposure definition	Exposure cut-off point	Accessment of cognitive function	Effect size and crude association results with 95%CI highest vs. lowest category	Effect size and adjusted association results with 95%CI highest vs. lowest category	Adjustment	Follow- up, yr	Quality scores
Takeuchi et al (2017) [30]	Japan	1566	Cohort	≥60	Number of teeth present	≥20, 10–19, 1–9, 0	DSM-III R	3.83 (2.47–5.93)	1.63 (0.95–2.80)	Sex, age, occupation, education, hypertension, diabetes mellitus, history of stroke, alcohol intake, tooth brushing frequency, regular visits to the dentist, and denture use.	5	16
Stewart et al (2015) [31]	Sweden	697	Cohort	70–92	Number of teeth present	≥25, 21–24, 9–20, 0–8,	DSM-III R	–	1.62 (0.84–3.11)	Age, education, social class, and vascular risk factors	37	13
Batty et al (2013) [30]	20 Countries	11,140	Cohort	55–88	Number of teeth present	≥22, 1–21, 0	MMSE	–	1.48 (1.24–1.78)	Age, sex, socio-economic CVD risk factors, treatment allocation and ethnicity	5	14
Yamamoto et al (2012) [32]	Japan	4425	Cohort	≥65	Number of teeth present	≥20, ≤19, 0	Standardized questionnaire	3.42 (1.05–11.08)	1.41 (0.42–4.70)	Age, adjusted household income, BMI, present illness, alcohol consumption, exercise, and forgetfulness	4	15
Paganini-Hill et al (2012) [33]	USA	5468	Cohort	52–105	Number of teeth present	26–32, 16–25, 1–15, 0	MMSE	0.84 (0.67–1.06)	–	–	18	13
Arrivé et al (2011) [34]	France	405	Cohort.	66–80	Number of teeth missing	< 11 ≥11	DSM-III R	1.35 (0.81–2.25)	–	–	15	12
Kim et al. (2007) [35]	Korea	686	Cohort	≥65	Number of teeth present	≥28 25–27, 15–24, 1–14, 0	DSM-IV	1.38 (1.12–1.69)	1.26 (1.00–1.59)	Age, gender and education, reported diet, vascular disease/risk, BMI and MAC, albumin and cholesterol	2.4	14
Stein et al (2007) [26]	USA	101	Cohort	75–98	Number of teeth present	10–28, 0–9	MMSE	2.69 (1.07–6.73)	2.20 (1.10–4.50)	Age, education, and apolipoprotein E4 allele	12	13
Shimazakil et al (2001) [36]	Japan	517	Cohort study	≥65	Number of teeth present	>20, 1–19, 0	Historical diagnosis information from medical records	5.20 (2.00–13.10)	2.40 (0.90–6.50)	Age, and classification of institution, physical health status, and cerebrovascular disorder	6	13

Notes: *BMI* Body mass index, *CI* Confidence interval, *CVD* Cardiovascular disease, *DSM-IV* The Diagnostic and Statistical Manual of Mental Disorders Fourth Edition, *DSM-III R* Diagnostic and Statistical Manual of Mental Disorders Third Edition, Revised, *MAC* Mid arm circumference, *MMSE* The Mini-Mental State Examination

**Table 3:** Summary of case-control studies included in the meta-analysis

Author / Year	Country	Sample size	Study design	Age, yr	Main exposure definition	Exposure cut-off point	Accessment of cognitive function	Effect size and crude association results with 95%CI highest vs. lowest category	Effect size and adjusted association results with 95%CI highest vs. lowest category	Adjustment	Quality scores
Gil-Montoya et al (2015) [37]	Spain	409	Case-control	> 50	Number of teeth present	20–32, 10–19, 1–9	DSM-IV	1.76 (1.05–2.95)	1.25 (0.67–2.36)	Age, sex, clinical attachment loss, oral hygiene habits, and hyperlipidemia	13
Gatz et al (2006) [27]	Sweden	3373	Case-control	59–107	Number of teeth missing	All, Half, Has all teeth	Clinical diagnostic evaluations for dementia	1.74 (1.35–2.24)	1.49 (1.14–1.95)	Age, sex, education, mentally stimulating activities, physical exercise, parents’ social class, short adult height	12
Kondo et al (1994) [38]	Japan	180	Case-control	43–89	Number of teeth missing	More than half of the teeth, Total denture with no own teeth	DSM-III R	1.90 (1.00–3.60)	–	–	11

Notes: *DSM-IV* The Diagnostic and Statistical Manual of Mental Disorder, Fourth Edition, *DSM-III R* Diagnostic and Statistical Manual of Mental Disorders Third Edition, Revised

**Fig. 2:**
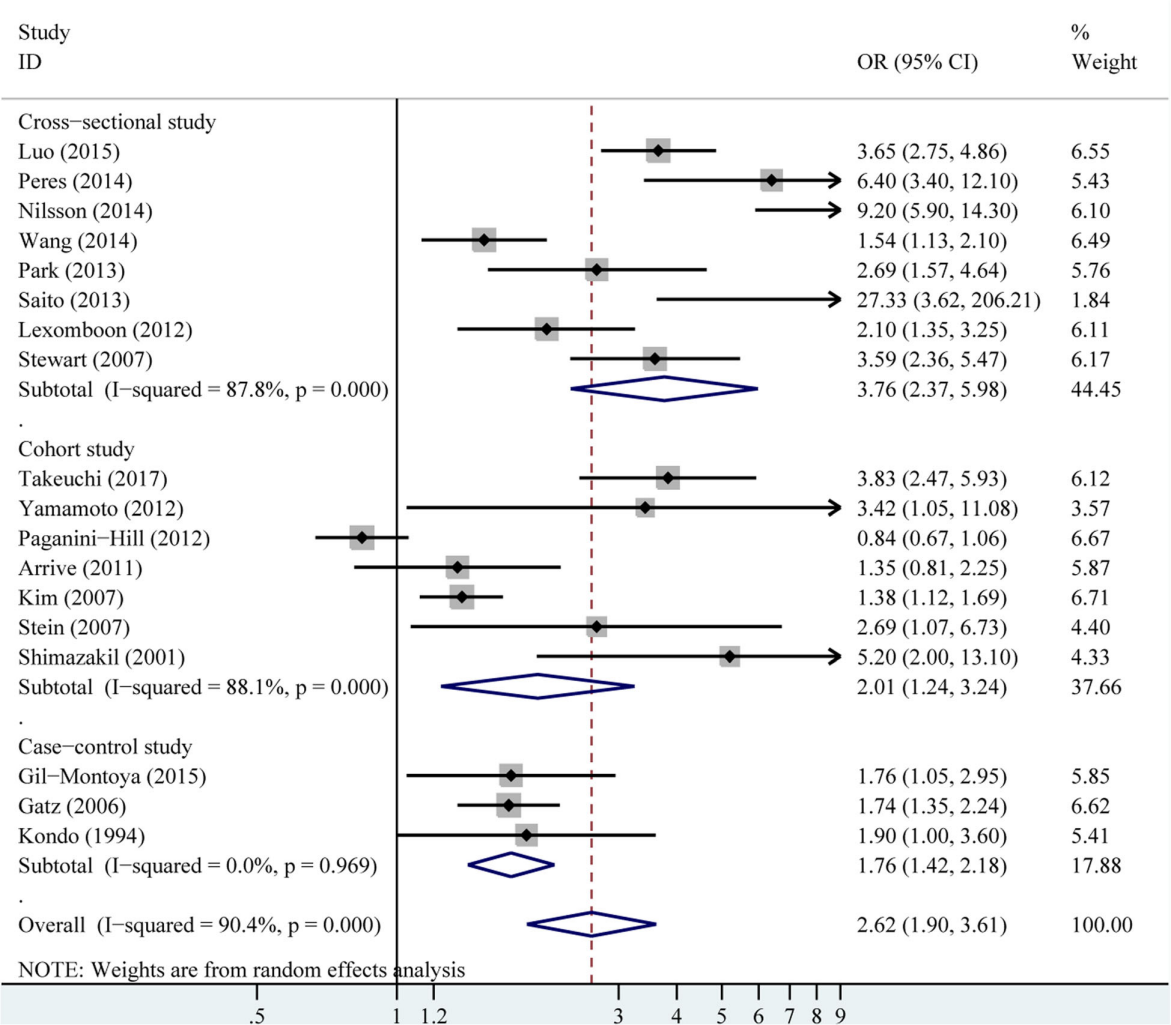
Pooled effect of crude results of tooth loss on dementia risk

**Fig. 3:**
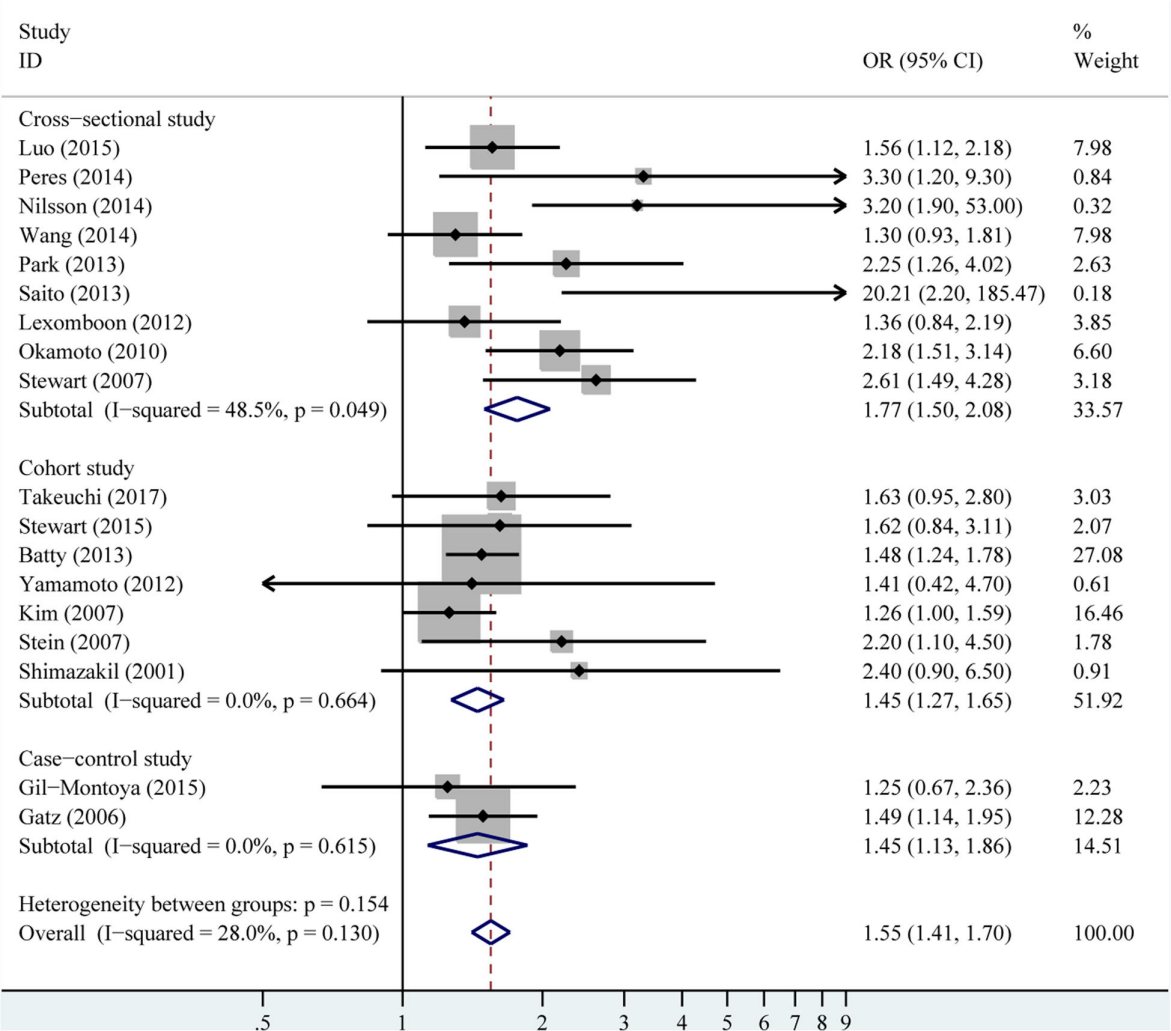
Pooled effect of adjusted results of tooth loss on dementia risk

There were 18 studies provided crude estimates for the risk of dementia. The pooled crude results revealed that patients with fewer tooth remaining had higher incidence of dementia (OR 2.62, 95% CI 1.90–3.61), with significant heterogeneity among these studies (*P* < 0.001, *I*^2^ = 90.40%), as shown in Fig. 2. The random-effects model was used for the crude results. The heterogeneity was also explored by subgroup and meta-regression analysis for the crude model (Table 4). The study design and sample size explained about 16.52% and 6.90% of the heterogeneity, respectively. There were 18 studies presenting adjusted estimates for the risk of dementia. The adjusted results remained significant when only adjusted results were pooled (OR 1.55, 95% CI 1.41–1.70), without obvious heterogeneity (*P* = 0.13, *I*^*2*^ = 28.00%; Fig. 3).

**Table 4:** Random-effect meta-analyses of tooth loss and dementia risk by subgroup and meta-regression analyses

	Studies with crude results			
	Number of estimates	Pooled OR and 95% CI	*P*-value	% heterogeneity explained
Study design				16.52
Cross-sectional	8	3.76 (2.37–5.98)	< 0.001	
Cohort	7	2.10 (1.24–3.24)	< 0.001	
Case-control	3	1.76 (1.42–2.18)	0.969	
Sample size				6.90
>1000	8	3.26 (1.79–5.93)	< 0.001	
<1000	10	1.95 (1.51–2.52)	0.008	
Study region				0
Asia	9	2.73 (1.83–4.07)	< 0.001	
Europe	6	2.57 (1.50–4.41)	< 0.001	
America	3	2.38 (0.57–9.91)	< 0.001	
Cognitive assessment				0
MMSE	8	3.12 (1.58–6.18)	< 0.001	
Others	10	2.38 (1.73–3.27)	< 0.001	
Total	18	2.62 (1.90–3.61)	< 0.001	–

**Fig. 4:**
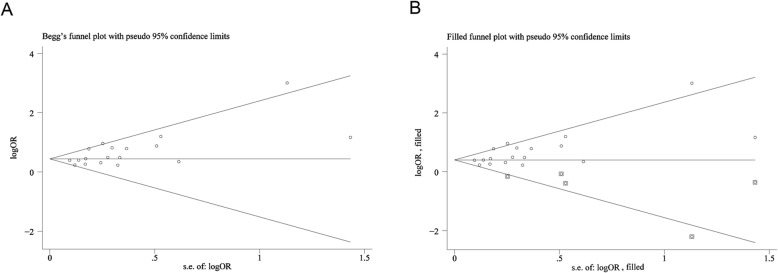
Funnel plots without and with Trim and Fill**. a** Begg’s funnel plot with pseudo 95% CIs of the adjusted model. **b** Filled funnel plot with pseudo 95% CIs of the adjusted model

Both the Begg-Mazumdar test (*P* = 0.11) and Egger’s regression test (*P* = 0.07) showed no significant evidence of publication bias for all included studies in the crude model (Additional file 1: Figure S1 A-B). Although the Begg-Mazumdar test showed not statistically significance (*P* = 0.15), the Egger’s regression test (*P* = 0.01) revealed significant publication bias in the adjusted mode (Additional file 1: Figure S1 C-D). Therefore, the trim and fill method was conducted as a sensitivity analysis by imputing hypothetical negative unpublished studies conservatively to mirror the positive studies that cause the funnel plot asymmetry [50–52]. The symmetrical funnel plot appeared with the imputed studies and the pooled analysis remained significant, incorporating the hypothetical studies in the adjusted model (OR 1.50; 95% CI 1.36–1.64; *P* < 0.001; Fig. 4).

## Discussion

Findings from this well-designed meta-analysis of 21 observational studies add to the accumulating evidence that tooth loss is a risk factor for dementia. Results from the crude model showed an overall 162% increase in dementia risk in adults, comparing individuals with high number of tooth loss to those with low number of tooth loss. We also observed an overall 55% increase in dementia occurrence risk in the adjusted model.

In the subgroup analysis by study design, the results remained significant in both the crude model and the adjusted model. However, it is possible to observe that in the crude model the association was not noted in European studies in the subgroup analysis by study region, while it was significant in Asia studies and American studies. These findings could be partially explained by the difference of healthcare systems and dental care access among different countries as described in the previous study [40]. Indeed, the great needs for dental care have been unmet in older adult population in many countries [9].

There is no known effective management for dementia and oral diseases are pretty common worldwide, particularly among older adults. Both dementia and tooth loss can result in significant impacts on people’s quality of life. Our findings have highlighted that adults living with higher number of tooth loss may have higher risk of dementia. In the general population, a general lack of knowledge of the importance of oral health partially account for the prevalence of tooth loss. Given the importance of tooth loss in the incidence risk of cognitive decline, oral health knowledge education programs and medical insurance policies are in urgent need among older adults population [9]. Oral health care and oral hygiene education are encouraged for both patients and their caregivers. Importantly, clinicians should be aware of this association, and oral examination should be a part of comprehensive assessments for those with high risk of dementia. Timely intervention of tooth loss may infuse new hopes for decreasing the incidence of dementia.

This study is not free of limitations. Firstly, we included cross-sectional studies in this analysis. In the light of such limitation, we conducted subgroup analysis by study design and the relationship remained significant in cohort studies, cross-sectional studies, and case-control studies. Secondly, different cognitive assessments were administered to determine participants’ cognitive function and various categories of the number of tooth loss were shown in studies. Finally, there was significant heterogeneity across studies in the crude model and publication bias in the adjusted model. Therefore, we used a random-effects model throughout to incorporate heterogeneity into the current analysis and we further identified possible sources of heterogeneity through meta-regression analyses. Additionally, the trim and fill analysis showed that the overall imputation did not alter the general results, indicating the results were robust to the possibility of unpublished negative studies. Regardless of the limitations, our review presents strengths that should be pondered. To the best of authors’ knowledge, this is the first well-designed systematic review with meta-analysis revealing both the crude and adjusted association between tooth loss and risk of dementia occurrence in adults. Secondly, the included studies from different settings demonstrate that the association between tooth loss and dementia risk is a global concern. Thirdly, the large number of sample size included in this analysis decreased the sampling error to a great extent.

## Conclusions

This review provides valuable evidence for the positive association between tooth loss and increased risk of dementia in adults. The association remained significant in both the crude and adjusted models. These findings may implicate clinically on improving oral health and cognitive function. However, considering the inherent limitations of the included studies, further well-designed longitudinal studies exploring the direct and indirect relationship between tooth loss and dementia are urgently needed for a more definitive conclusion.

## Additional file

Additional file 1: ** Figure S1.** Begg’s funnel plots and Egger’s publication bias plots**.** (A-B) Begg’s funnel plot and Egger’s publication bias plot of the unadjusted model, respectively. (C-D) Begg’s funnel plot and Egger’s publication bias plot of the adjusted models, respectively. (TIF 117 kb).

## Abbreviations

AMTS: Abbreviated mental test score
BMI: Body mass index
CES-D: The Center for epidemiologic studies depression scale
CI: Confidence interval
CVD: Cardiovascular disease
DSM-III R: Diagnostic and Statistical Manual of Mental Disorders Third Edition, Revised
DSM-IV: The Diagnostic and Statistical Manual of Mental Disorders Fourth Edition
HR: Hazard ratio
IL-1: Interleukin-1
IL-6: Interleukin-6
MAC: Mid arm circumference
MMSE: Mini-Mental State Examination
OR: Odds ratio
RR: Risk ratio
TNF-α: Tumor necrosis factor –α
TMIG-IC: The Tokyo Metropolitan Institute of Gerontology Index of Competence
