# Supplementary figures and images for: Tooth loss as a risk factor for dementia: systematic review and meta-analysis of 21 observational studies
Source: BMC Psychiatry. 2018 Oct 20;18:345. doi: 10.1186/s12888-018-1927-0 (PMC6195976; doi:10.1186/s12888-018-1927-0)

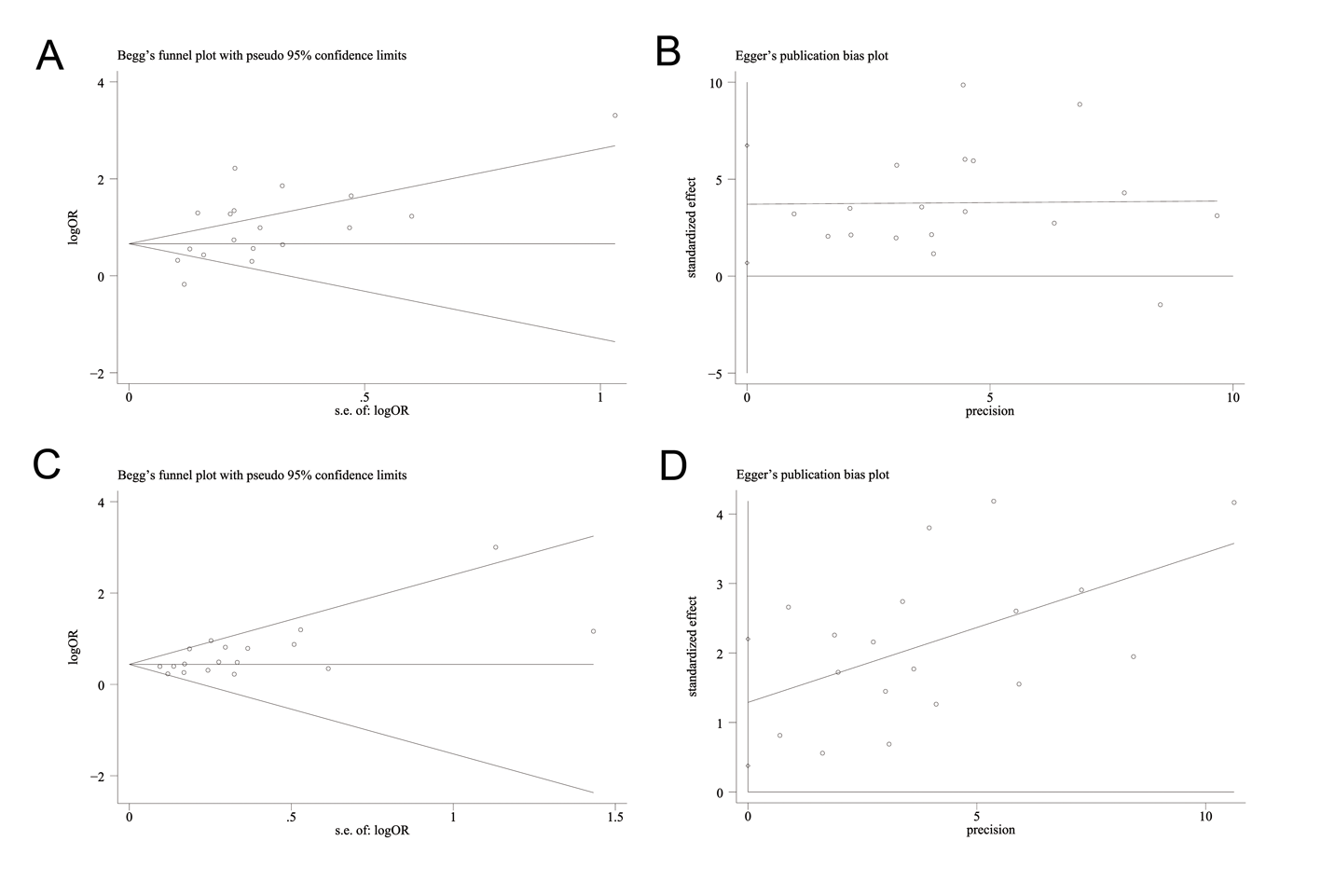

Supplement: Supplementary file 1 — Figure S1. Begg’s funnel plots and Egger’s publication bias plots. (A-B) Begg’s funnel plot and Egger’s publication bias plot of the unadjusted model, respectively. (C-D) Begg’s funnel plot and Egger’s publication bias plot of the adjusted models, respectively. (TIF 117 kb). [file 12888_2018_1927_MOESM1_ESM.tif]
